# MoNa – A Cost-Efficient, Portable System for the Nanoinjection of Living Cells

**DOI:** 10.1038/s41598-019-41648-6

**Published:** 2019-04-02

**Authors:** Matthias Simonis, Alice Sandmeyer, Johannes Greiner, Barbara Kaltschmidt, Thomas Huser, Simon Hennig

**Affiliations:** 1 Biomolecular Photonics, University of Bielefeld, Universitätsstr. 25, 33615 Bielefeld, Germany; 2 Department of Cell Biology, University of Bielefeld, Universitätsstr. 25, 33615 Bielefeld, Germany; 3 Molecular Neurobiology, University of Bielefeld, Universitätsstr. 25, 33615 Bielefeld, Germany; 4 Institute of Biophysical Chemistry, Hannover Medical School, Carl-Neuberg-Str 1, 30625 Hannover, Germany

## Abstract

Injection techniques to deliver macromolecules to cells such as microinjection have been around for decades with applications ranging from probing whole organisms to the injection of fluorescent molecules into single cells. A similar technique that has raised recent interest is nanoinjection. The pipettes used here are much smaller and allow for the precise deposition of molecules into single cells via electrokinetics with minimal influence on the cells’ health. Unfortunately, the equipment utilized for nanoinjection originates from scanning ion conductance microscopy (SICM) and is therefore expensive and not portable, but usually fixed to a specific microscope setup. The level of precision that these systems achieve is much higher than what is needed for the more robust nanoinjection process. We present Mobile Nanoinjection (MoNa), a portable, cost-efficient and easy to build system for the injection of single cells. Sacrificing unnecessary sub-nanometer accuracy and low ion current noise levels, we were able to inject single living cells with high accuracy. We determined the noise of the MoNa system and investigated the injection conditions for 16 prominent fluorescent labels and fluorophores. Further, we performed proof of concepts by injection of ATTO655-Phalloidin and MitoTracker Deep Red to living human osteosarcoma (U2OS) cells and of living adult human inferior turbinate stem cells (ITSC’s) following neuronal differentiation with the MoNa system. We achieved significant cost reductions of the nanoinjection technology and gained full portability and compatibility to most optical microscopes.

## Introduction

Methods for delivering molecules to single cells have become a standard tool in the biosciences following the introduction of microinjection in 1979 by Feramisco and Kreis *et al*.^1,2^. The method facilitates the delivery by pressure-driven injection of biomolecules into cells via small hollow glass capillaries. To date, several single cell delivery methods have been developed, utilizing e.g. photothermal effects^3^ or electrokinetics for accumulation and delivery of charged molecules into the nucleus of cells, using a small charged lance^4,5^.

Based on previously existing scanning ion conductance microscopy (SICM) systems^6^, first attempts of the delivery of molecules with nanopipettes via electrokinetics were recently achieved^7–9^ resulting in a new technique called nanoinjection. The nanoinjection technique combines the advantages of traditional hollow glass pipette approaches and electrokinetics. Here, the almost unlimited reservoir of molecules from microinjection and the controllable electrokinetic-driven delivery in combination with a precise axial position feedback system provides a method for the fast and easy positioning of the pipette and insertion of molecules - e.g. fluorescent probes - into single living cells. Exploiting the unique approach technique by tracking the ionic current flowing between two electrodes through the tip of the nanopipette, this allows for the precise axial positioning of the nanopipette with respect to the plasma membranes of single cells. This enables the location-specific penetration of the membrane and injection of single cells as well as cellular compartments such as the nucleus with molecules. Nanoinjection was shown to date to be able to deliver up to three different intracellular fluorescent stains, which were mixed and filled into the nanopipette, stepwise by applying different voltages during a single injection step. But in principle the delivery of a higher number of fluorescent probes within a single injection step should be possible. Also, instant super-resolution imaging directly after the injection process is possible^9^ with the nanoinjection technique. The use of small pipette tip diameters of approx. 100 nm ensures the high survival rates of the injected cells of 92% accompanied with normal proliferation behavior^10^.

SICM systems achieve high positioning resolutions of <10 nm in axial and <50 nm in lateral direction combined with spatial control of the scanning probe tip in the sub-nanometer range^11^ resulting in the fast and precise scanning of a surface. This is usually accomplished by expensive piezo-driven xyz-stages and high-end (i.e. high frequency and high resolution) z-piezo stacks which control a nanopipette-oscillation during the scan. To measure the ionic current with adequate signal to noise ratio and frequency, a microelectrode amplifier/pre-amplifier configuration accompanied by a lock-in amplifier is commonly found on those setups providing excellent current detection. All of the equipment mentioned above is heavy, difficult to set up and thus lacks flexibility to connect with different workspaces without major adjustments and days of calibration.

However, injecting mammalian cells by nanoinjection does not require very fast and precise control of the nanopipette or a very high sensitivity for measuring ionic currents, as the size of adherent mammalian cells varies but is usually not smaller than about 8 µm in diameter. To approach a single cell by a nanopipette, lateral control within a range of 0.5 to 1 µm and axial control of the nanopipette in the range of 20 to 50 nm is sufficient. Additionally, tracking of the ionic current within a range of <2 nA with a temporal resolution of approx. 100 ms allows for the precise approach of the injection probe to the target. Thus, we use custom-built hardware and refrain from employing elaborate, expensive xyz-piezo stage and microelectrode amplifier systems. By developing a nanoinjection system based on these parameters we were able to drastically reduce the size, weight and cost of all our components, resulting in a cost-efficient, slim and portable system, which can be attached to almost any inverse optical microscope with small effort (Supplementary Figs S7 and S8**)**. Nevertheless, our Mobile Nanoinjection (MoNa) system still achieves very high axial precision of ~3 nm compared to the ~0.7 nm of our SICM setup. The custom-built electrode amplifier operates on noise-levels approximately ten times higher than the commercial setup (~82 pA compared to ~8 pA). We show that this is still sufficient enough to provide the positional feedback needed for nanoinjection to indicate the penetration of a cell and nuclear membrane through the change in the ion current.

## Results

**Figure 1:**
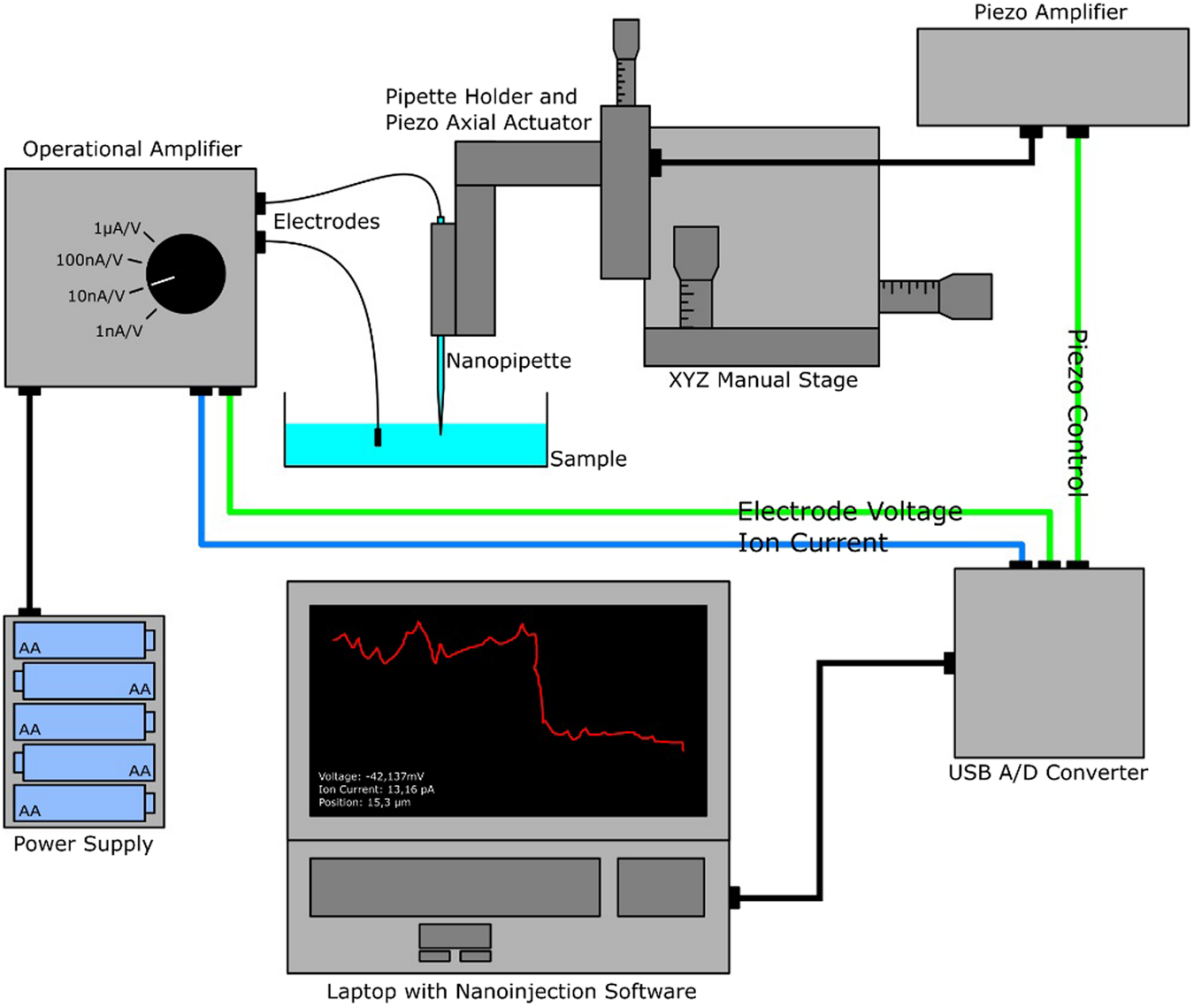
Schematics of the mobile nanoinjection setup. The entire system is monitored and synchronized with a small USB data acquisition interface controlled by our nanoinjection software. Two analog output channels (green wires) are used to provide voltage for the electrodes (channel AO 0) and to control the pipette movement (channel AO 1) via a piezo actuator. The ion current is amplified by a custom operational amplifier and converted according to a selectable range to ±10 V to then be sent to the analog input channel (AI 0) of the USB A/D converter (blue wire). As a power supply for the amplifier, either a conventional power supply or a 15 V battery pack can be used to gain additional flexibility.

The principles of nanoinjection are described in several recent publications^9–12^. Electrodes are placed inside a nanopipette that is drawn to an approx. 100 nm tip diameter and the cell medium, respectively (see Fig. 1). Controlling the voltage, probe molecules can then be precisely deposited from the pipette via (di-) electrophoresis or osmotic flow. The ionic current is used for positional feedback of the nanopipette and – in combination with the detected wide-field fluorescence – for monitoring the injection process. As the pipette tip is slowly lowered towards the cell membrane, a distinct drop in the ion current can be observed. The same happens for the nuclear membrane of a cell, which enables the precise and reliable injection of molecules into the cytoplasm as well as into the nucleus of a single cell.

The MoNa system (Fig. 1) – or any similar nanoinjection setup – can be divided into three main components: 1. Voltage control and ion current measurement; 2. nanopipette movement and positioning; 3. monitoring and synchronization of the injection setup as a whole.

In our original SICM setup used for nanoinjection^9^, the ion current measurement is performed by an Axopatch 200B Amplifier in combination with a CV 203BU Headstage, (Molecular Devices, California, USA). Here, we replaced the common microelectrode amplifier/pre-amplifier configuration by a simple custom-built operational amplifier (Supplementary Figs S5 and S6). Four selectable current ranges: ±10 nA, 100 nA, 1 µA and 10 µA can be chosen with consequent measurement precision determined by their respective noise levels. 15 V DC are needed to operate the amplifier and can be provided by a power supply or a battery pack for additional portability. The output to the data acquisition interface is scaled over the entire range from −10 V to +10 V. A full characterization of the input/output voltage behavior is depicted in Supplementary Fig. S2.

The nanopipette holder (Supplementary Fig. S6) is attached to a commercial xyz linear stage (ULTRAlign Precision XYZ Linear Stage, Newport Corporation, California, USA) with a lateral and axial positioning precision of 100 nm (depending on the screws used in this linear stage). This stage is used for manual lateral positioning and coarse axial control of the nanopipette. Connected to the linear stage is a second linear stage, driven axially by a piezo micrometer screw actuator (P-854.00, Physik Instrumente, Germany) with a total range of 25 µm and sub-nanometer accuracy, to enable the approach and injection process. For comparison, our SICM system works with a xyz piezo microscope positioning stage (Nano-PDQ375HS, Mad City Labs, Wisconsin, USA) that moves the sample itself.

The control interface of the MoNa system consists of a small 14 bit USB device capable of measuring and providing voltages of ±10 V ($1bit:\,\frac{20V}{{2}^{14}-1}=1,221mV$) with a sample speed of 20 kHz (USB-6001, National Instruments, Texas, USA). It provides the voltage for the electrodes, as well as performing the approach process and monitoring the ion current. Combined with the piezo actuator input range of 0 V to +10 V this leads to a minimal step size of ~3.1 nm ($\frac{25\mu m}{10V}\times 1,221mV=3,052nm$). The accuracy of the current measurement depends on the selected range of the amplifier and ranges from approx. 0.061 pA to 61 pA. Compared to the corresponding ion current noise levels, which are about three orders of magnitude higher during a typical approach process, these values are negligible. The two outputs (piezo actuator and electrode voltage) and one input (ionic current) are controlled and displayed by purpose-written software (see Supplementary Fig. S4) running in LabVIEW (National Instruments) on a standard notebook equipped with an Intel i3 processor and 4 GB RAM, compared to a high performance I/O card (PCIe-6251, National Instruments) running on a desktop PC equipped with an i5 processor and 8GB RAM for control of our original SICM setup. Pipette position, injection speed and electrode voltage can easily be controlled with the mobile setup while simultaneously monitoring the real-time acquisition of the ion current (Supplementary Fig. S3).

Comparing the cost of our complete setups, we were able to save roughly 85% of the cost of the high-end system (Tables 1 and S1). We were using some fairly old and/or second-hand equipment which makes a precise comparison difficult, but considering the technical requirements it should be easy to find appropriate alternative options.

**Table 1:** Comparison of the MoNa and SICM-based injection systems.

Component	Original SICM Setup	MoNa Setup
Ion current measurement, voltage control	Axopatch 200B Amplifier + CV 203BU Headstage (Molecular Devices)	Custom build operational amplifier
Pipette movement (manual)	ULTRAlign Precision XYZ Linear Stage (Newport)	ULTRAlign Precision XYZ Linear Stage (Newport)
Pipette movement (automated)	Nano-PDQ375HS + Nano-Drive 85 (Mad City Labs)	P-854.00 (Physik Instrumente) Piezo Driver P-863 (Physik Inst.)
Analog I/O	NI PCIe-6251 + 2 x BNC-2090 + 2 x SHC68-68-EPM Shielded Cable (National Instruments)	USB-6001 (National Instruments)
Software	LabVIEW Base Development System (National Instruments)	LabVIEW Base Development System (National Instruments)
**Additional component**		
Pipette Puller	Sutter Instruments P2000	Sutter Instruments P2000

The main components are compared separately. (The used piezo driver P-854.00 was second hand and approx. 20 years old. Current alternative: Piezo amplifier E-836 (Physik Instrumente)).

**Figure 2:**
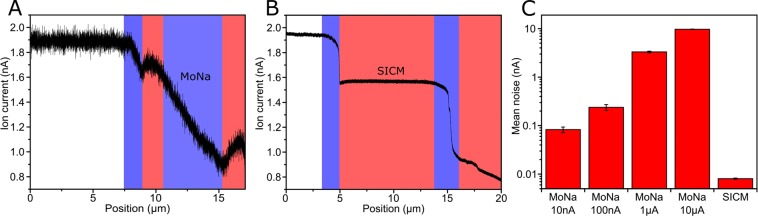
Comparison between the ion current during approach of a cell and the noise levels of the MoNa and SICM system **(A**,**B)** Ion current during approach and penetration of an U2OS cell with the MoNa system (current range: ±10 nA) (A) and the SICM system (**B**). The *blue area* indicates decreasing current, as the pipette tip approaches and comes in contact with the membranes. After penetration, the current stops dropping notably and enters the *red area* with anything between a slight up or down slope of ion current. This principle can be observed for both outer cell membrane and nuclear membrane. Although the signal acquired by the commercial setup is much smoother and contains less noise, the injection features are clearly visible in the current data of our system as well. For both experiments, the electrode voltage was set to +100 mV and the approach speed was ~1 µm/s. **(C)** Mean noise (RMS, root mean square) levels and standard deviations of different range settings of the operational amplifier and for comparison the original SICM (pre-) amplifier configuration. These values cover the entire signal chain from electrodes to amplifier to A/D converter to computer. The measurements were performed under typical approach conditions i.e. phosphate buffered saline (PBS) was used as medium and the applied voltages ranged from −200 mV to +200 mV in 100 mV increments. As expected, the SICM system delivered the lowest noise level at (8.08 ± 0.24 pA). The custom setup gives (82 ± 11 pA), (239 ± 34 pA), (3.31 ± 0.17 nA) and (9.68 ± 0.12 nA) dependent on the selected current range.

To compare the capabilities of our custom-built mobile nanoinjection system with our original nanoinjection setup based on a SICM setup, we first determined the noise levels of the entire signal chain that is used to measure the ion current. For each setting, 10,000 samples were recorded at 1 kHz sample speed for five different voltages in the range of ±200 mV, which represents typical settings for an injection approach. Phosphate buffered saline was used as a medium inside the pipette and counter-electrode bath. The coarsest setting of the custom amplifier (10 µA) yields a noise level of (82 ± 11) pA. This is about ten times more than the original system with (8.08 ± 0.24) pA (Fig. 2C).

Next, we compared the ionic current feedback while penetrating a human osteosarcoma cell (U2OS) at the position of the nucleus. The pipette is approached automatically towards the cell membrane, while continuously monitoring the ion current. The SICM principle^6^ shows a decrease in the current observed at the plasma membrane of the cell, followed by a small plateau as the tip has pierced the outer membrane and entered the inside. A second decrease and plateau indicates penetration of the nuclear membrane and insertion of the tip into the nucleus. The apparent widths of these features vary from cell to cell but are always observable. The low noise level of the SICM system results in nice and easily distinguishable penetration phases (Fig. 2B). However, with our MoNa design and an at the most tenfold higher current noise (Fig. 2C), the typical injection features can also be determined without any problem (Fig. 2A). With this sufficiently accurate feedback, we are able to selectively place the pipette tip into the cytoplasm and the nucleus of single living adherent cells. An example of the approaching pipette, approached and inserted into the cytoplasm of a living U2OS cell can be found in Supplementary Movie S1.

**Figure 3:**
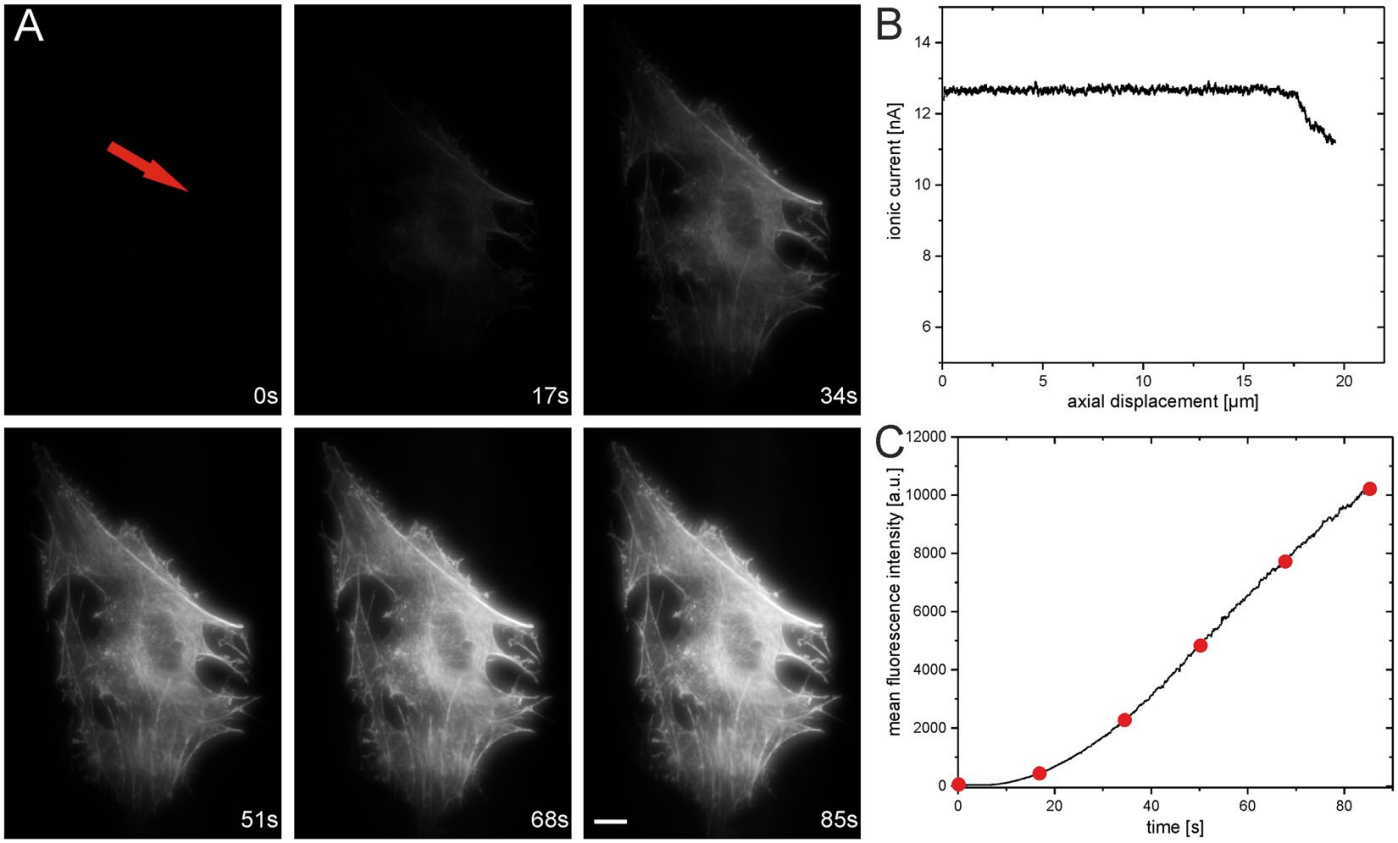
Nanoinjection of ATTO655-Phalloidin into the cytoplasm of a single living adult human inferior turbinate stem cell (ITSC) after neuronal differentiation (the injection point is indicated by the red arrow). (**A**) Fluorescence view of the injection of ATTO655-Phalloidin molecules into the cell with an injection voltage of 200 mV applied for 85 s. The images show the delivery of the functionalized fluorescent molecules into the cell by increased fluorescence. The fluorescence is confined within the cell and an instant binding to the actin structure is visible. After ~50 s, the actin structure of the living cell is clearly visible under the applied imaging conditions. The injection was carried out with a concentration of 10^−5^ M ATTO655-Phalloidin solved in PBS inside the nanopipette. (**B**) Approach curve of the nanopipette towards the cell. The tip of the nanopipette was placed manually ~20 µm above the coverslip surface. After 17 µm of approach, a first decrease of the ionic current is visible, indicating the contact of the tip with the cell. The approach was stopped manually at 19 µm to avoid breaking of the nanopipette. Afterwards the injection of fluorescent probes began. (**C**) Average fluorescence intensity of single images taken over the period of injection, indicating the delivery of molecules into the cell. The red dots indicate the time points of the respective fluorescence views in a. Scale bar: 5 µm, images were taken at low wide-field illumination conditions with an integration time of 120 ms.

**Figure 4:**
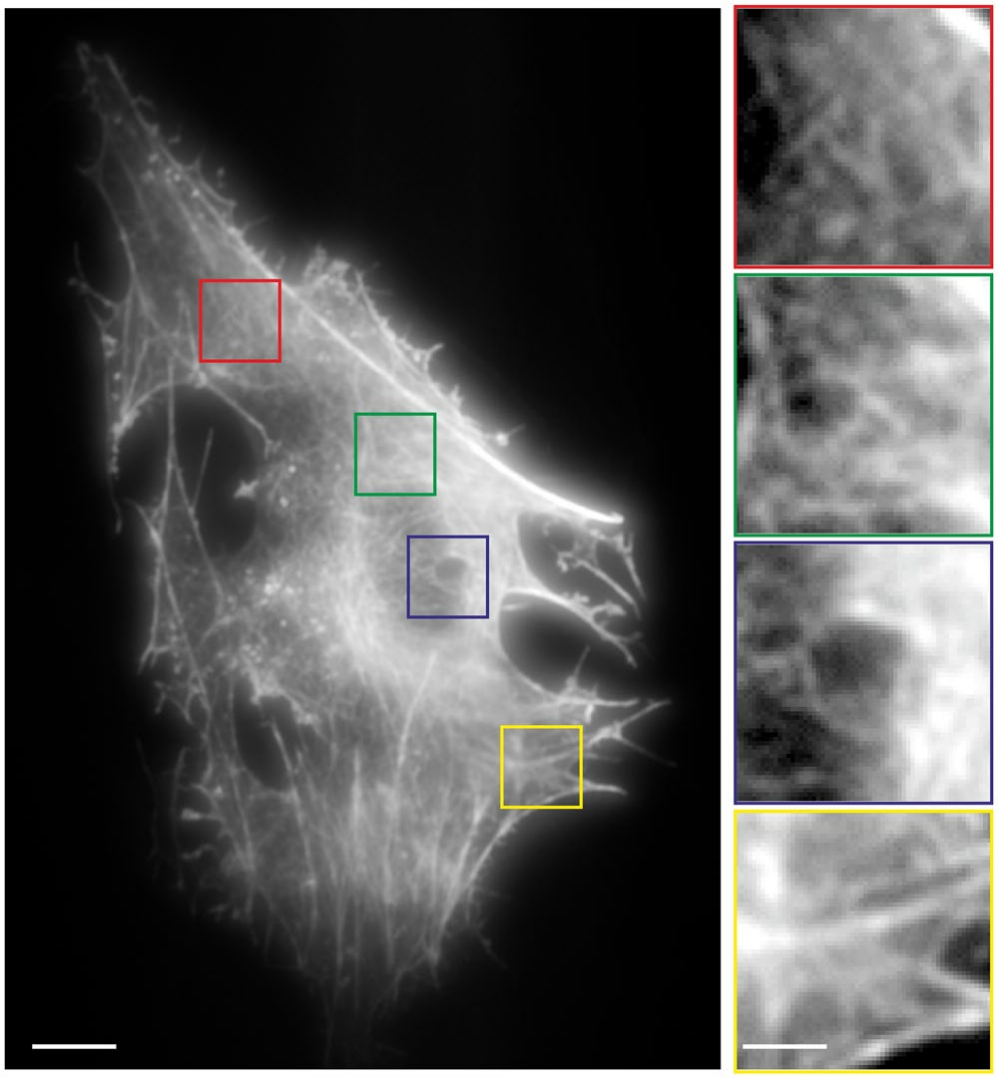
Detailed view of the labeled ITC (Fig. 3) after the injection of ATTO655-Phalloidin. The insets of the indicated areas show nicely the actin finestructure, being visible in each part of the cell and indicating an even distributed labeling over the whole actin structure of the cell. Scale bar: 5 µm, inset: 1.5 µm.

To show the capabilities of the MoNa system, we attached the MoNa system to a standard inverse wide-field fluorescence microscope (Olympus IX71) equipped with an EMCCD camera and a laser with an excitation wavelength of 647 nm for excitation of the fluorophores. First, we injected ATTO655-Phalloidin (Atto-Tec Siegen, Germany) to visualize the actin structure of a single living cell by injection of the functionalized molecules into the cytoplasm. For this purpose, the nanopipette was approached to the cytoplasm of a living adult human inferior turbinate stem cell (ITSC) following neuronal differentiation. After approaching and penetrating the plasma membrane the labeling begins by increasing the voltage to 200 mV. Figure 3A shows the fluorescence view of the labeling process. After 85 s, the injection procedure is completed and the fully labeled actin structure of the cell is visible. In contrast to fixed cell labeling of cells with ATTO655-Phalloidin, the fine structure is not destroyed due to fixation reagents and is nicely visible within the cell (see Fig. 4**)**.

In a second test, we injected Mito Tracker Deep Red (Thermo Fisher Scientific, Germany) into a single living U2OS cell. Although Mito Tracker is a cell permeant fluorescent probe for live cell imaging, we were able to label the mitochondria of just one living cell within an ensemble of cells by nanoinjection and prevent significant diffusion of the probe to neighboring cells. We were also able to observe mitochondrial dynamics for at least 30 min by wide-field fluorescence imaging with low illumination intensity and a temporal resolution of 120 ms, by continuously injecting the cell with the fluorescent probes. For this purpose, we filled the nanopipette with a stock solution of 10^−3^ M of Mito Tracker Deep Red dissolved in DMSO and applied an injection voltage of 2.5 V after completing the approach process. Due to the low conductivity of DMSO the molecules were leaving the pipette hesitantly, extending the initial labeling of the cell to approx. 12 min. Nevertheless, tracking of a subset of mitochondria was possible during this time (see Supplementary Fig. S1 and Movie S2).

**Figure 5:**
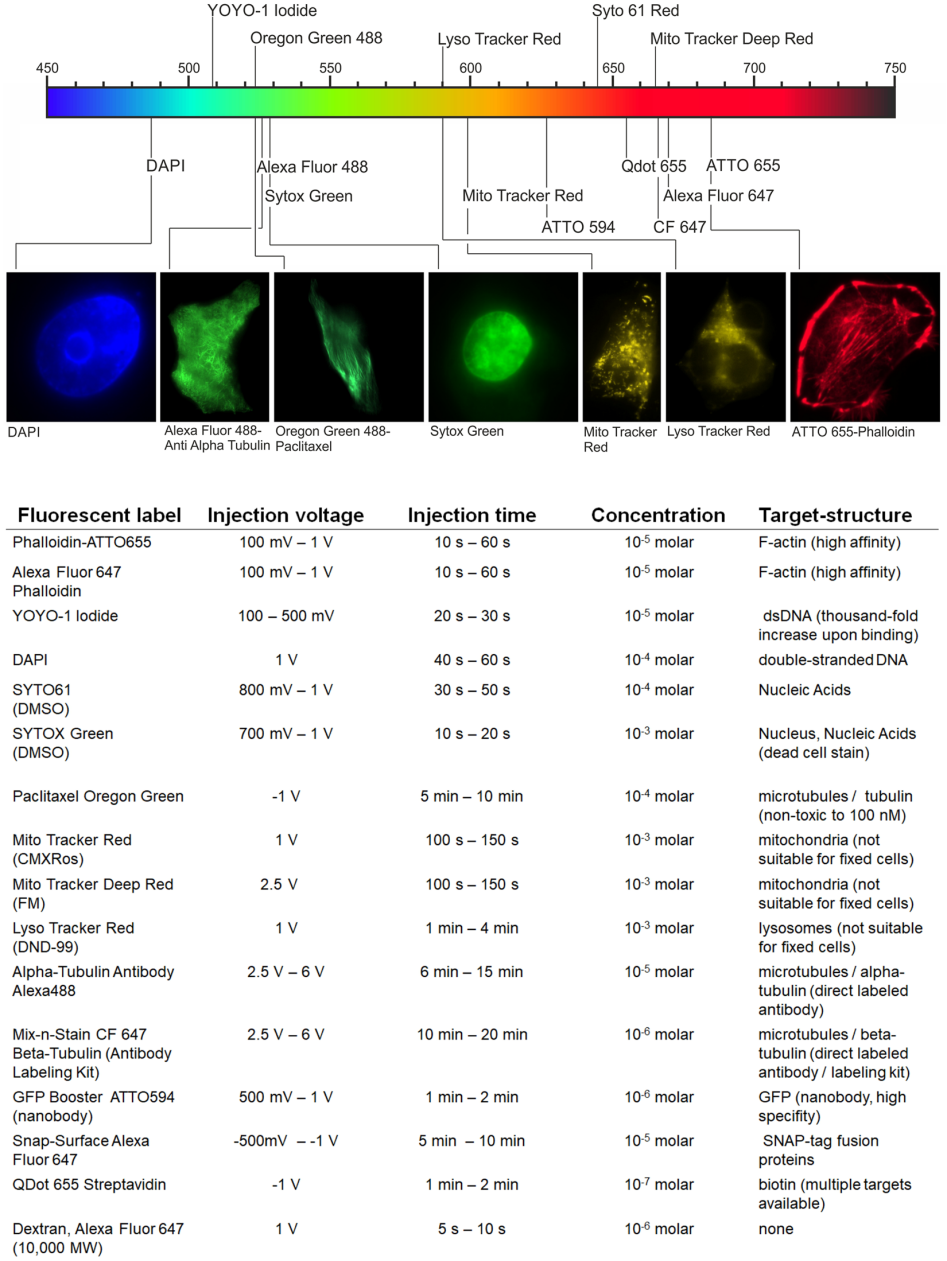
Overview of fluorescent probes and fluorophores which were successfully tested. The injections were performed under PBS conditions (nanopipette and bath) at 37 °C, unless mentioned separately. The injection time was defined by the start of the injection till the complete fluorescent staining of the cellular lumen or organelle.

Nanoinjection is not limited to a specific type of fluorescent probes or fluorophores. In principle, every molecule of appropriate size and solubility can be injected via nanoinjection. But as the fluorescence signal provide instant feedback about successfully delivered molecules, injection without fluorescence-based feedback is not easy to realize if not even completely impossible. Therefore, a fluorescently labeled molecule is strongly recommended to confirm the injection of molecules. Besides the fluorescent feedback, one has to consider the injection time and the injection voltage needed for a specific molecule. In our experience injection times and -voltages differ a lot from molecule to molecule and additionally depend strongly on the buffer conditions. In general the injection of molecules is limited by too high injection voltage and/or long injection times, which will consequently lead to serious cell damage or directly to apoptosis^10^. Figure 5 shows a list of all fluorescent probes and fluorophores that have been so far tested successfully in combination with nanoinjection. The respective injection conditions are based on our experience and worked well for our injection procedure and cell lines (mostly U2OS and Cos7 cells). The injection was tested with a standard 100 nm diameter nanopipette using PBS (pipette- and bath solution) at 37 °C. Otherwise it is noted in the table. Overall, 16 different fluorescent probes and fluorophores were tested, including quantum dots, nanobodies and antibodies.

## Discussion

Manipulation of single cells is limited to only a few experimental approaches. The standard single cell manipulation tool is the well-known microinjection technique. But limitations of this technique e.g. the relatively large tip size of 500 nm and the approach mechanism, which relies on a priori calculating the distance between pipette tip and cell surface and not on a real-time feedback during the approach of the tip, are major drawbacks. By employing the current-feedback system of the SICM principle for single-cell manipulation and tacking into account the smaller tip sizes of nanopipettes, which is typically 100 nm in diameter, one can overcome the drawbacks of microinjection. But as SICM systems require expensive and inflexible equipment for current detection and sample adjustment, this turns out to be a major drawback of manipulation systems based on the SICM principle. Although the amplifier of the MoNa system turns out to provide a noise level which is roughly tenfold higher compared to the amplifier of our original SICM setup, we were able to reliably approach single cells and perform injection of fluorescent probes. The sample approach with a minimal step size of 3.1 nm turns out to be sufficient for the approach of single cells. Furthermore, taking the total height of typical eukaryotic cells into account, even an axial step size of 25 to 50 nm should be acceptable to visualize an approach curve and thus reliably approach and penetrate single cells. The lateral sample adjustment turns out to be more difficult in comparison to our original setup, as the target cell has to be moved to the center of the field of view in a first step with the xy stage of the microscope. Then, in a second step, the pipette has to be adjusted manually to the desired injection point of the cell. The precision of the manual positioning screws turned out to be sufficient for cytoplasmic as well as for nuclear injection. However, the original SICM system with the nanopipette laterally fixed at the center of the field of view and a movable piezo-driven sample stage was easier to handle and provided more accurate adjustment of the exact injection point.

Differences during the injection of fluorescent probes using the MoNa system were not observed. Here the injection voltages of 200 mV for ATTO655-Phalloidin and 2.5 V for mitotracker deep red respectively was appropriate to deliver the fluorescent molecules into the cell, visualizing the targets of interest. As most of the patch-clamp or electrode amplifiers used for SICM are only providing a voltage of at most ±2 V, the amplifier of the MoNa system has the advantage to provide a voltage range of ±10 V. This would allow e.g. a faster injection of fluorescent molecules into the target. Also, approaches in a less conductive environment (e.g. directly in cell medium) as it is typically provided and used by the PBS conditions would be possible.

ATTO655-Phalloidin and mitotracker deep red were chosen as examples for nanoinjection within this paper. Besides this, we showed the compatibility of a wide range of fluorescent probes for nanoinjection including the respective injection conditions.

Based on the tested probes, we identified 2 main groups of molecules by sorting the fluorescent probes with respect to their injection voltage and injection time:

1. Low injection voltage in combination with short injection times (typically in the range of ±1 V and below 1 min).
2. High injection voltage and/or long injection times (typically between 5 and 8 V and between 3 and 15 min)

We assume, that group 1 represents small molecules, which have an intrinsic charge and therefore direct electrophoretic forces drive the molecules from the nanopipette into the cell leading to injection times below 1 min with injection voltages within the range of ±1 V. If the molecules don´t show an overall charge, the transfer is driven either by dielectrophoretic forces, osmotic flow or a combination of both^13^. In general, this type of injection seems to be associated with a voltage above/below of ±1 V in combination with longer overall injection times, corresponding to group 2, whereby group 2 also seems to be affected by the molecular weight, which then results in a significant increase of the injection time.

## Conclusions

By resorting to inexpensive components, we were able to develop a working nanoinjection system that has the same injection capabilities as our original SICM-based setup. With this new system, we could realize cost reductions of about 85% compared to a full-blown SICM instrument (excluding the cost of a pipette puller device). Furthermore, the design of the new device is small, lightweight, flexible and fits into a large suitcase including all of its components. MoNa is easy to use and after moving the system, it is possible to start working within two hours due to minimal installation and calibration effort. We demonstrated the utility of this system by injecting fluorophores into living transformed as well as primary cells, which represent hard to access and difficult to manipulate^14^ targets. Using the nanoinjection technique, we were able to address and manipulate these cells directly by fluorescent probes.

The provided list of fluorescent probes which were successfully tested for nanoinjection shows the compatibility of nanoinjection to a wide range of fluorophores and probes. Although, the list is far from being complete, it provides researchers with a starting point to develop an injection strategy for their specific experimental conditions.

Most parts of the MoNa system are exchangeable with equipment already at hand or available second-hand. For example, the piezo actuators could be replaced by cheaper step-motors and as long as the pipette holder is sufficiently robust, one could also use a different design. To make things easy on the software-side, we used a commercially available low-cost USB A/D converter that is controllable by a broad variety of languages and programming environments.

The system can be combined with almost every available inverted optical microscope, by using generic mounting plates. Thus, it is possible to take advantage of a variety of commercial systems e.g. to perform long-time nanoinjection experiments and maintain ideal cell culture conditions throughout. Another interesting experiment would be the combination with other cost-efficient, compact setups, such as low-cost *d*STORM super-resolution microscopy systems^15,16^. This way an affordable super-resolution nanoinjection system could be set up fairly easy. Especially for smaller laboratories this could be a great option as it is simple to build and maintain.

## Methods

### Cell Preparation

Human bone osteosarcoma cells (U2OS) were grown in Dolbecco’s Modified Eagle Medium (DMEM) with 5% fetal bovine serum (FBS) added and cultivated at 37 °C in a humidified 5% CO_2_ atmosphere. For all experiments, cells were transferred to standard LabTeks (Lab-Tek II Chamber Slide System, Nunc) at the desired density and given at least 24 h to settle down.

### Isolation and culture of adult human inferior turbinate stem cells (ITSCs)

Isolation, culture and differentiation of ITSCs was performed as described previously^17–19^. Briefly, human nasal inferior turbinates were extracted via routine nasal surgery after informed consent according to local and international guidelines (Bezirksregierung Detmold/Münster; Declaration of Helsinki). Isolation and all further experimental procedures/methods were ethically approved by the ethics commission of the Ärztekammer Westfalen-Lippe and the medical faculty of the Westfälische Wilhems-Universität (Münster, Germany) (approval reference number 2012- 15-fS). All methods used in the present study were performed in accordance to local and international guidelines (Bezirksregierung Detmold/Münster; Declaration of Helsinki). Extraction of human nasal inferior turbinates was followed by subsequent mechanical disintegration and enzymatical dissociation of the tissue. ITSCs were isolated by pre-cultivation of the dissociated tissue in Dulbecco’s modified Eagle’s medium/Ham’s F-12 (1:1) (DMEM/F-12; Sigma-Aldrich, Germany) with penicillin and streptomycin (0.1 mg/ml penicillin, 100 U/ml streptomycin; Sigma-Aldrich), amphotericin B (0.25 mg/ml; Sigma-Aldrich), L-glutamine (200 mM; Sigma-Aldrich), epidermal growth factor (EGF; 20 ng/mL; Peprotech, Germany), basic fibroblast growth factor (bFGF-2; 40 ng/ mL; Miltenyi Biotec, Germany), B27 supplement (labmade) (hereinafter referred to as ‘standard medium’) and heparin (0.5 U/ml, Sigma-Aldrich) in a humidified incubator at 37 °C, 5% CO_2_ and 5% O_2_. Isolated ITSCs were expanded in standard medium supplemented with 10% human blood plasma (Institut für Laboratoriums- und Transfusionsmedizin, Bad Oeynhausen, Germany). For neuronal differentiation, ITSCs were cultivated on Lab-Tek™ chamber slides (Thermo Fisher Scientific) in DMEM (Sigma-Aldrich) containing 10% FCS (Sigma-Aldrich). After 48 hours, 1 μM dexamethasone (Sigma-Aldrich), 2 μM insulin (Sigma-Aldrich), 500 μM 3-isobutyl-1-methylxanthine (Sigma-Aldrich), 200 μM indomethacin (Sigma-Aldrich) and 200 mM ethanol were added to the medium. Medium was changed twice a week by replacing half of the medium volume and additionally supplemented with 0.5 μM retinoic acid (Invitrogen) and N2 supplement (Invitrogen) after seven days of differentiation. Injections of ITSCs were performed after 14 days of differentiation.

### Injection

Living U2OS or ITSCs cells were plated on the microscope at room temperature. Prior to the measurements, DMEM medium was replaced with pre-warmed PBS to increase the conductivity of the medium. Nanopipettes were pulled and filled with the injection solution. The setup and injection process was carried out as described elsewhere^8^.

## Supplementary information

Supplementary Information

Nanopipette approach

Injection of Mitotracker to a single cell

Nanoinjection executive program for LabView

## Data Availability

All data generated and/or analyzed during this study are included in this published article and its supplementary information files.
