# Supplementary material for: MoNa – A Cost-Efficient, Portable System for the Nanoinjection of Living Cells
Source: Sci Rep. 2019 Apr 2;9:5480. doi: 10.1038/s41598-019-41648-6 (PMC6445100; doi:10.1038/s41598-019-41648-6)
Supplement: Supplementary file 1 — Supplementary Information [file 41598_2019_41648_MOESM1_ESM.docx]

MoNa – A Cost-Efficient, Portable System for the Nanoinjection of Living Cells

*Matthias Simonis ^1^, Alice Sandmeyer ^1^, Johannes Greiner ^2^, Barbara Kaltschmidt ^2,3^, Thomas Huser ^1^, and Simon Hennig ^4^ **

*^1^* Biomolecular Photonics, University of Bielefeld, Universitätsstr. 25, 33615 Bielefeld, Germany

^2^ Department of Cell Biology, University of Bielefeld, Universitätsstr. 25, 33615 Bielefeld, Germany

*^3^* Molecular Neurobiology, University of Bielefeld, Universitätsstr. 25, 33615 Bielefeld, Germany

*^4^* Institute of Biophysical Chemistry, Hannover Medical School, Carl-Neuberg-Str 1, 30625 Hannover, Germany

**SUPPLEMENTARY INFORMATION**

*^*^* Corresponding Author: hennig.simon@mh-hannover.de

ADDITIONAL FIGURES


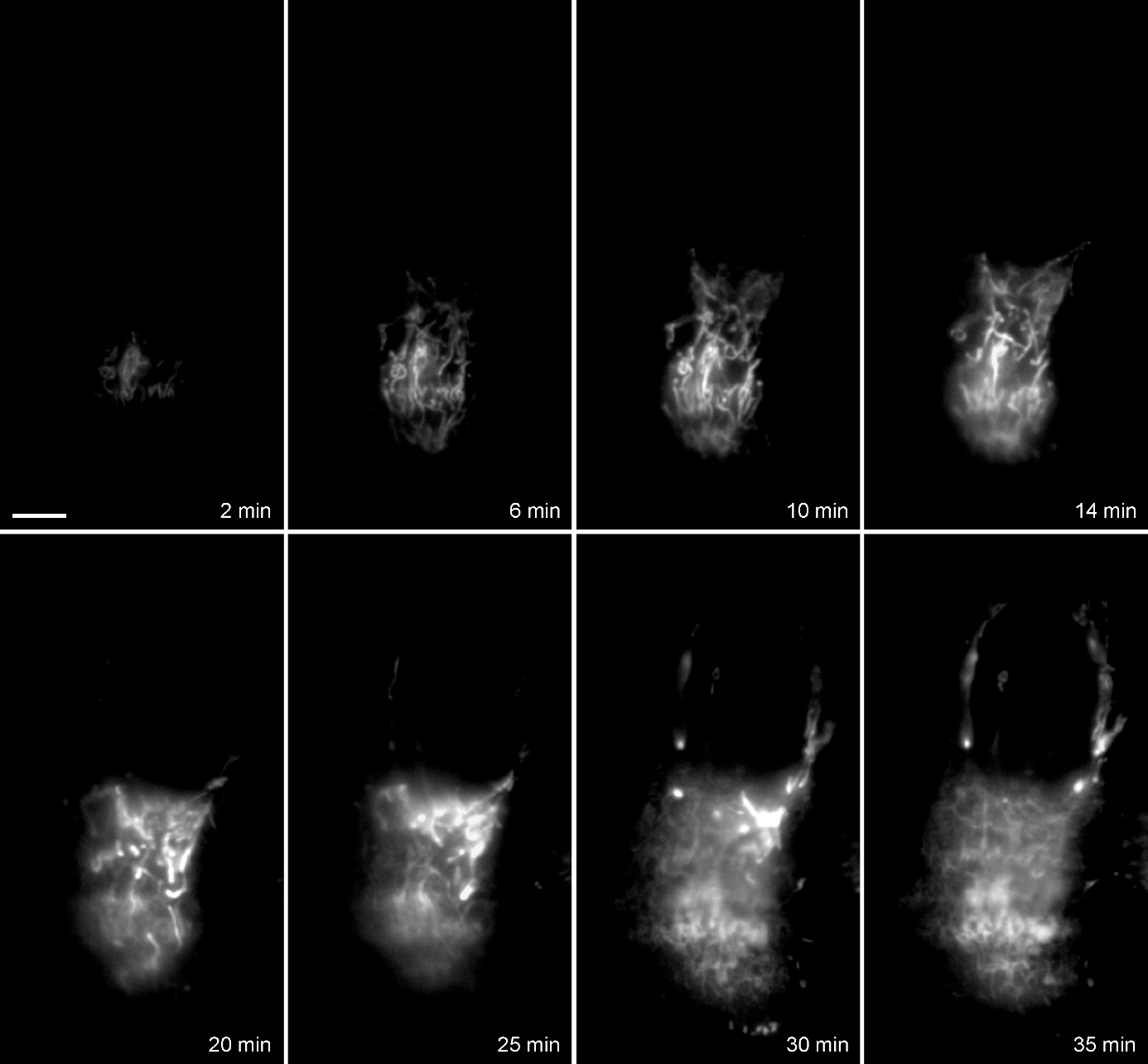


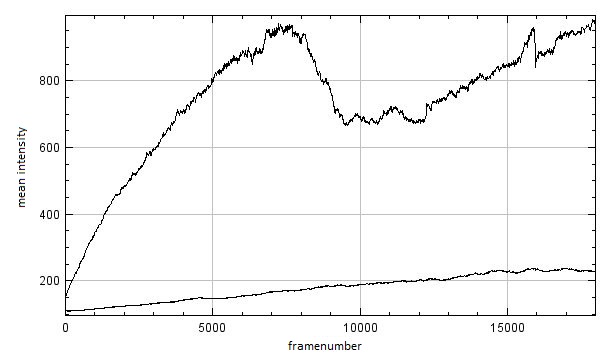


Target cell

Neighboring cell

**Figure S1.** Upper part: Nanoinjection of Mito Tracker Deep Red into the cytoplasm of a single living U2OS cell. Temporal representation of the injection of Mito Tracker molecules into the cell with an injection voltage of 2.5 V applied for 35 min. The fluorescence images show the delivery of the fluorescent molecules into the cell by increasing fluorescence. The nanopipette was filled with the stock solution of 10^-3^ M of Mito Tracker Deep Red dissolved in DMSO. The injection was performed with an injection voltage of 2.5 V. After 2 min first fluorescence of was visible indicating the mitochondria of the cell. Due to the low conductivity of DMSO the molecules were leaving the pipette hesitantly, extending the initial labeling of the cell to approx. 12 min. Nevertheless, tracking of a subset of mitochondria was possible during this time. After approx. 30 min the mitochondria structure broke down, indicating a stressed state of the cell. Scale bar: 5 µm, images were taken at low to normal wide-field illumination conditions with an integration time of 120 ms. Micrographs represents an average of 5 single images each. A full movie of the staining process can be found in the supplementary information (Movie S2). Lower part: Average fluorescence intensity of the target cell (direct injection of mitotracker fluorescent probes) and a neighboring cell, placed approx. 5µm from the cell membrane of the target cell. The graphs clearly show, that a minor fraction of mitotracker diffuses trough the cell membrane of the target cell and enters the neighboring cell. The fluorescence signal in the neighboring cell is continuously increasing, and reaches a maximum of ~20% of the fluorescence signal of the target cell.


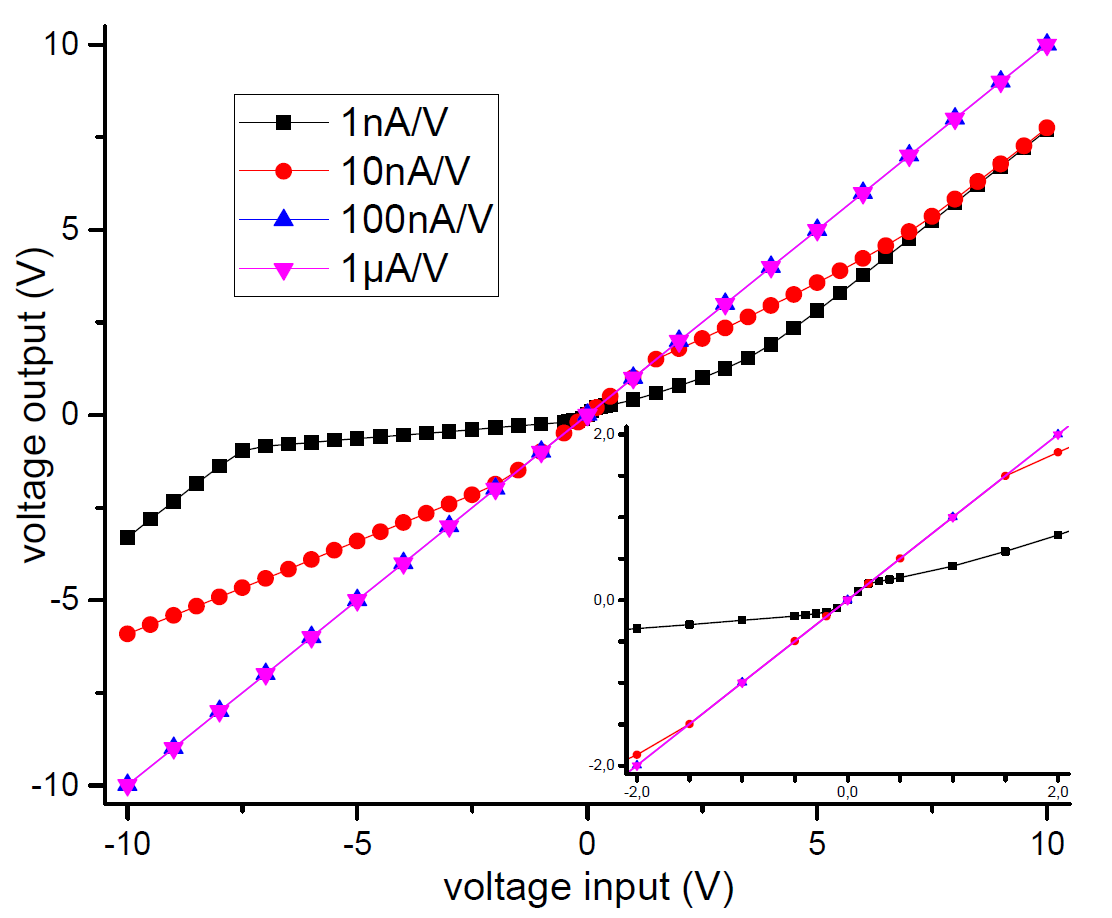


**Figure S2.** MoNa amplifier I/O voltage characterization. Behavior of the applied (input) voltage and the output voltage between the electrodes. The characterization was performed for each sensitivity range of the amplifier. The settings 1 µA/V and 100 nA/V show a linear dependency between input and output voltage. The settings 10 nA/V and 1 nA/V show a nonlinear behavior over the whole range, whereas the output voltage is always lower than the input voltage. Additionally the output voltages are asymmetric to zero.

SOFTWARE

The operator controls the axial position of the nanopipette and the applied voltage at the tip to deliver the molecules with a custom-built software. The source code is of the control software is provided as a .vi file, written in LabVIEW (version 14.0.1, National Instruments) and enables the communication and data acquisition between all devices **(Figure S3)**.

**Figure S3.** Scheme of the communication channels between the control devices. The red arrows display the output channels and the green arrows the input channels. To deliver molecules with the nanopipette, a voltage is applied at the tip by an amplifier. The amplifier can also readout the ionic current which is needed for the approach process. The axial position of the tip is controlled by a z-Piezo. The data acquisition is done by an USB A/D Converter and the software-controlling is done by a self-written LabVIEW program.

As mentioned in the “*Results"* section, an USB A/D converter (USB-6001, National Instruments) is used to apply, control and readout the voltage at the control devices. To control the applied voltage at the tip, a customized amplifier is used. The amplifier also converts the measured ionic current back into a voltage. In case of the axial position of the tip, it is only possible to apply a voltage to the z-Piezo but not to have a feedback about the position. Therefore, the software needs to have the ability to display the measured voltages and to give the possibility of control. Also, an automated approach of the tip towards the sample needs to be implemented.

To run all these devices correctly, the program is divided into three different parts: initialize, manual and stop. The initialize and stop part are only needed due to technical issues. During these parts, the axial position of the tip and the applied voltage is configured. In both cases, there is no voltage applied at the devices. The manual mode runs during the whole experiment. LabVIEW generates automatically a GUI for controlling which can be customized **(Figure S4)**.


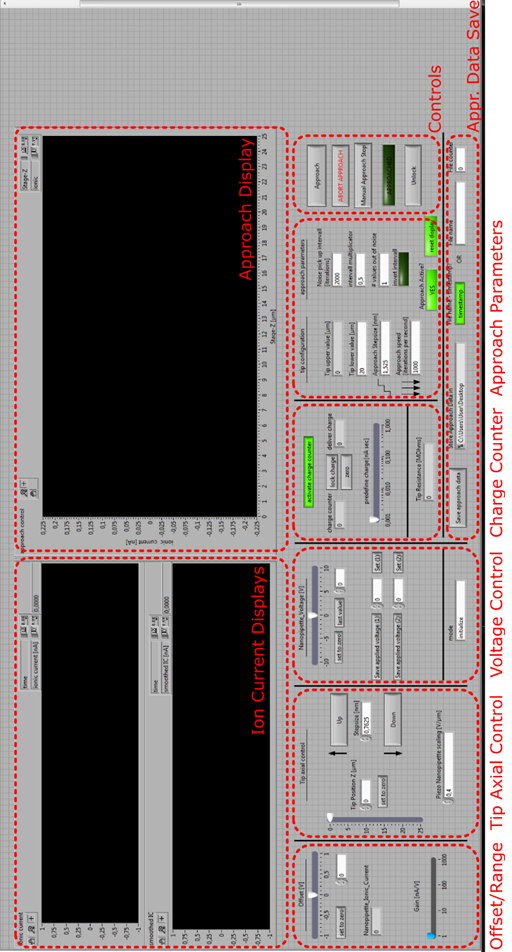


**Figure S4.** Screenshot of the Nanoinjection software designed in LabVIEW. The *Ion Current Displays* show the ionic current in real time. The raw data is displayed and in the other case 50 data points are averaged and then displayed. With *Offset/Range* a voltage can be set to counteract non-zero currents if no voltages are applied and the same gain as at the amplifier can be set. The tip position is monitored at *Tip Axial Control* and axial position can be defined here. With *Voltage Control* the applied voltage at the tip is controlled to deliver molecules. The *Charge Counter* is a tool to define a certain charge which is about to deliver. With *Approach Parameters* it is possible to set the speed of approaching and where the approach should stop. The ionic current during the approach is displayed at *Approach Display*. The button of *Control* was implemented to start and stop the approach. In case the ionic current during the approach should be saved, the operator can do this afterwards with *Appr. Data Save*.

To operate the approach correctly, some adjustments needs to be done before the actual measurement. Each electrode is unique and the experimental surrounding is different every time. Therefore, a different voltage at the electrodes is measured for each experiment, although the approach has not been started. To achieve a real offset of the voltage, a constant voltage needs to be applied at the beginning which can be done at the *Offset/Range* section. The ionic current is displayed at *Ion Current Display* in real time. The raw data is shown and also a smoothed curve, where 50 datapoints are averaged. Also, the same gain as at the amplifier needs to be set.

For the approach a voltage is applied at *Voltage Control* and the value can be set to zero or set to the last value. Even two values can be saved and applied afterwards for a more efficient workflow. Now, the operator starts the approach with the Approach-Bottom at *Controls* and the default values of *Approach Parameters* are appropriate. At *Tip Axial Control*, it the value of the axial position of the tip changes in real time and on the *Approach Display* the ionic current (raw and smooth) can be seen. In case, the tip should start at another position than the default value, the operator can set the axial position and the approach starts from this point. To stop the approach it is possible to define parameters, but normally the operator does this by eye to decide if the right position is achieved. In case there is a rapid decrease of the ionic current, the tip is in the cell or near a surface. At this point the approach should be stop with the Manual-Approach-Stop-Button. Now, a voltage, with a different sign, can be applied to deliver molecules. If enough molecules are delivered, the voltage is set to zero and the tip should be placed at the default position by the Unlock-Button. The data of the ionic current during the approach process can be saved afterwards at the *Appr. Data Save* section.

HARDWARE

**
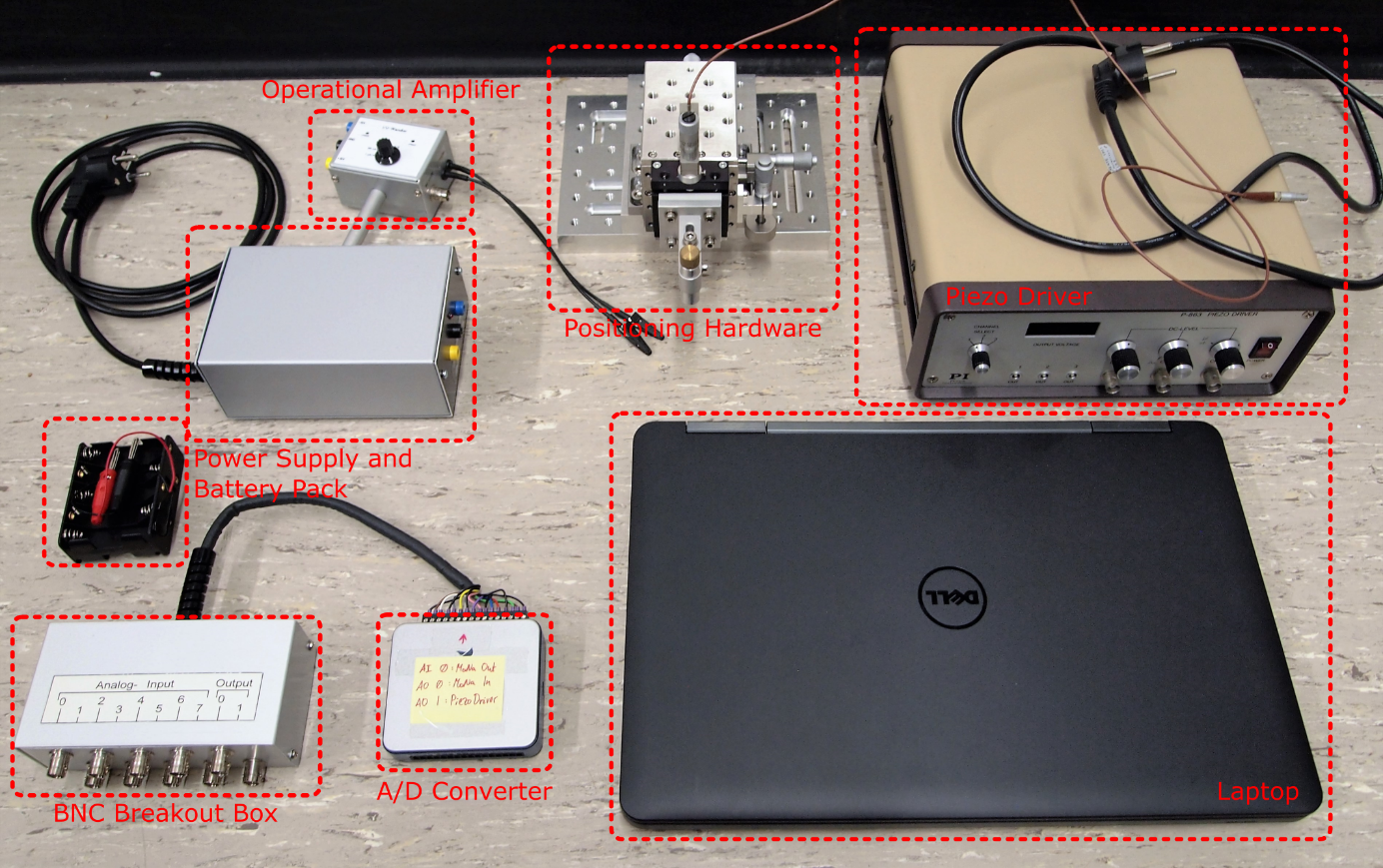

Figure S5.** Overview of all MoNa components (excluding misc. items like screws, cables, etc). Note that either a battery pack or a power supply (15V) can be used to power the Amplifier.

**
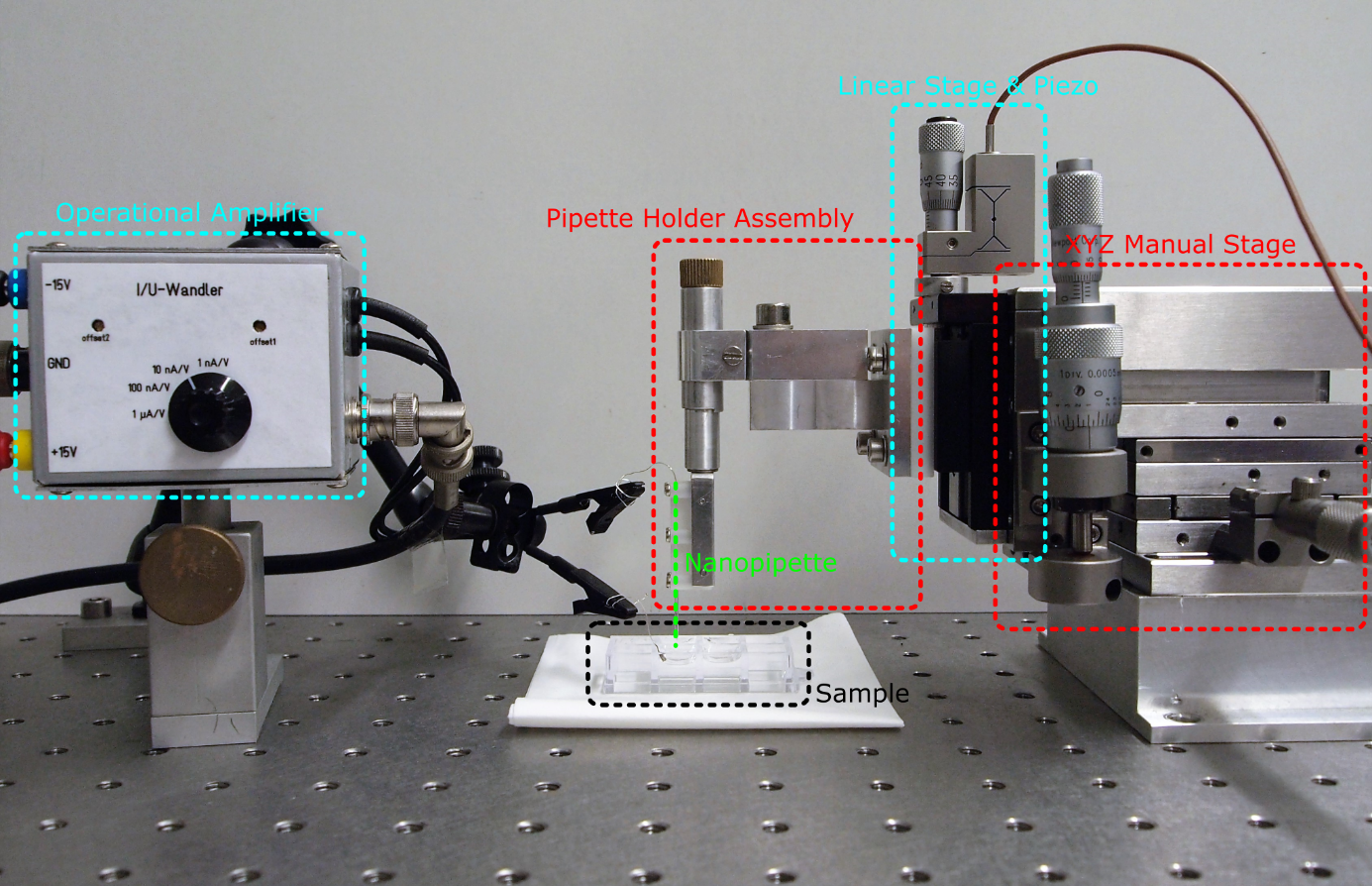
**

**Figure S6.** Close up of the main components of the MoNa System. The operational amplifier and piezo (cyan) are controlled by the USB A/D converter (not shown). The pipette holder and xyz manual stage (red) allow for coarse positioning of the nanopipette (green) above the sample (black).

EXAMPLES OF THE ATTACHED SYSTEM

**
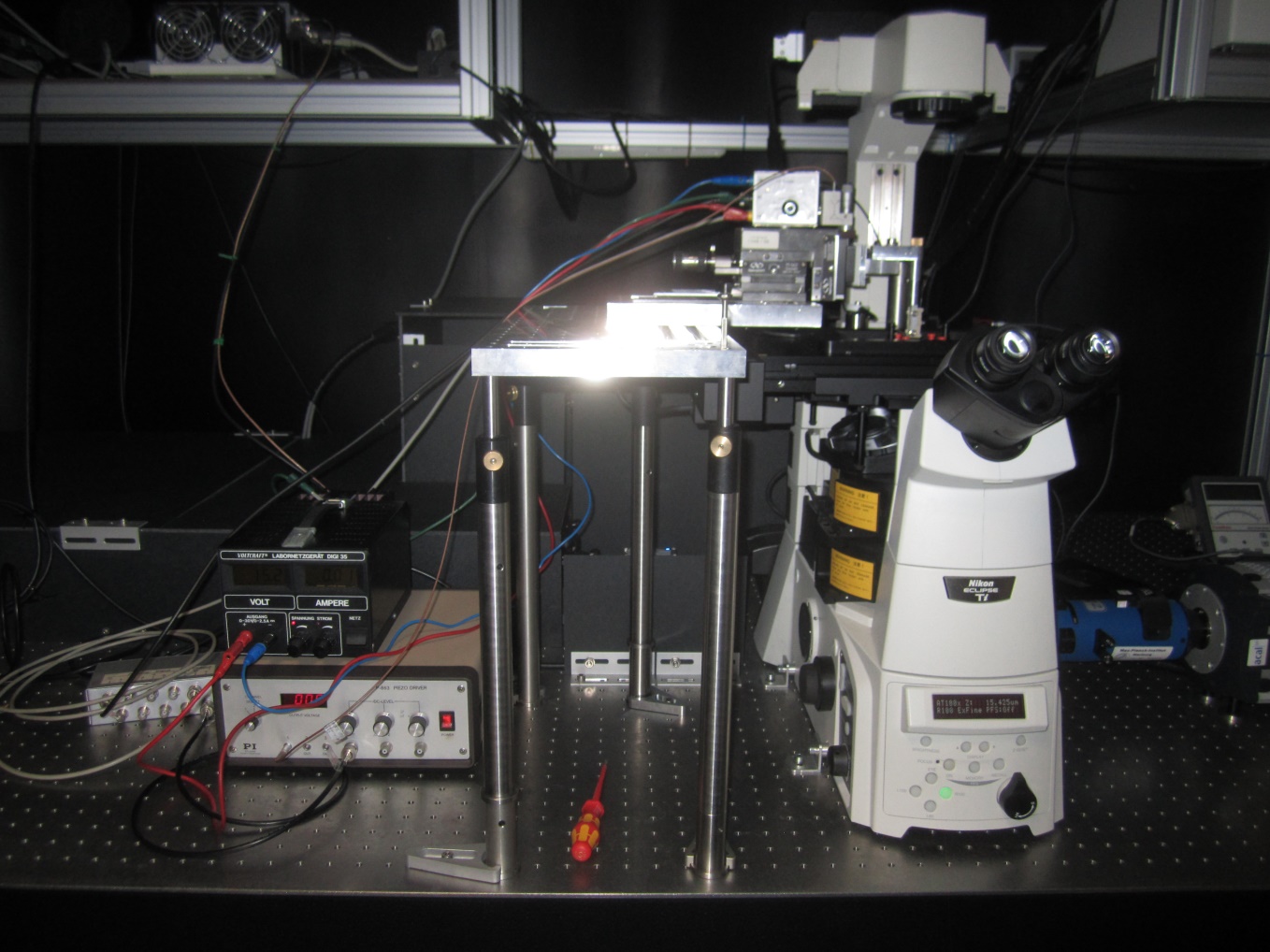
**

**Figure S7.** Examples of the attached MoNa system: The MoNa System attached to a Nikon eclipse TI. The System was attached to the microscope from the left side. The condenser of the microscope was removed for an optimized position of the pipette holder.

**
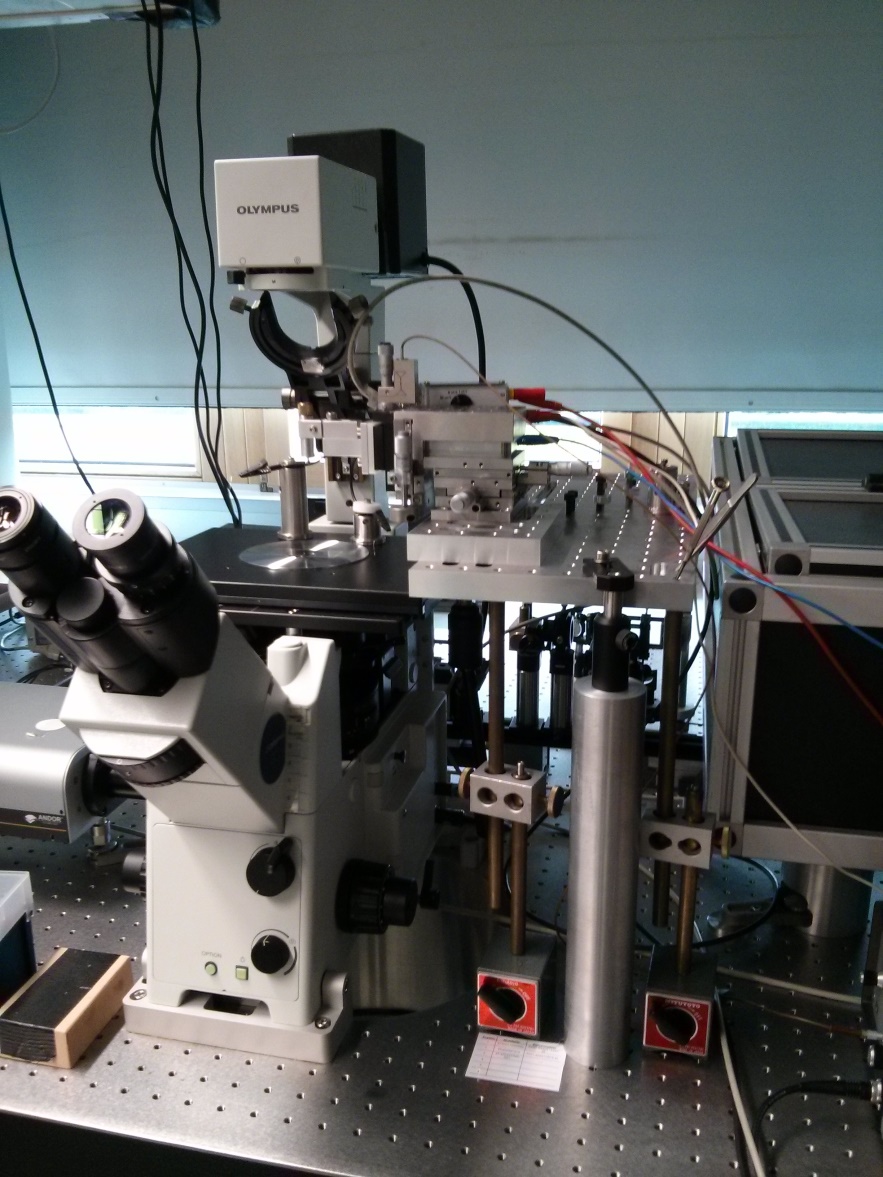
**

**Figure S8.** Examples of the attached MoNa system: The MoNa system attached to an Olympus IX71. The system was attached to the microscope from the right side. The condenser of the microscope was removed for an optimized position of the pipette holder.

**Table S1.** Rough price comparison of our two different injection systems. The main components are compared separately. All Prices are inclusive of German VAT (19%). As the driver is 20 years old, the prize was corrected for inflation. A current alternative: PIAK10 10 mm Travel Piezo Inertia Actuator + TIM101 Controller (Thorlabs, 1551,88 €)

|  | **Commecial SICM setup** | | **Cost-efficient MoNa system** | |
| --- | --- | --- | --- | --- |
| **Component** | **Part** | **Price** | **Part** | **Price** |
| Ion current measurement, voltage control | Axopatch 200B Amplifier  + CV 203BU Headstage (Molecular Devices) | 11,038.00 € (2004) | Custom build operational amplifier | ~ 40 € (2016) |
| Pipette movement (manual) | ULTRAlign Precision XYZ Linear Stage (Newport) | 2,256.24 € (2016) | ULTRAlign Precision XYZ Linear Stage (Newport) | 2,256.24 € (2016) |
| Pipette movement (automated) | Nano-PDQ375HS + Nano-Drive 85 (Mad City Labs) | 28,842.00 € (2007) | P-854.00 (Physik Inst.)  Piezo Driver P-863 (Physik Instrumente) | 675.00 € (2000)  ~ 819.00 € (1996) |
| Analog I/O | NI PCIe-6251 + 2 x BNC-2090 + 2 x SHC68-68-EPM Shielded Cable (National Instruments) | 3,374.82 € (2016) | USB-6001 (National Instruments) | 219.29 € (2016) |
| Software | LabVIEW Base Development System (National Instruments) | 1,013.29 € (2016) | LabVIEW Base Development System (National Instruments) | 1013.29 € (2016) |
| Miscellaneous (incl. PC/Laptop, cables, screws, holders, electrodes, etc.) |  | ~ 1000.00 € |  | ~ 1000.00 € |
| **Sum 1** (basic MoNa setup) |  | **47,524.35 €** |  | **6,022.82 €** |
| **Additional components for a ready-to-use nanoinjection setup** |  |  |  |  |
| Nanopipettes | Pipette Puller (Sutter Instruments: P2000) | 19,380.00 € | Pipette Puller (Sutter Instruments: P2000) | 19,380.00 € |
|  | Borosilicate cylindrical glass with filament (GB100F 8P, Science Products) | 11.30 € | Borosilicate cylindrical glass with filament (GB100F 8P, Science Products) | 11.30 € |
| Ion current | Ag wires for electrodes (AG-8T, Science Products) | 80.00 € | Ag wires for electrodes (AG-8T, Science Products) | 80.00 € |
|  | Counterelectrodes (E-205, Science Products) | 10.00 € (each) | Counterelectrodes (E-205, Science Products) | 10.00 € (each) |
|  | Sodium Hypochlorite Solution (Fisher, SS290-1) | 99.00 € | Sodium Hypochlorite Solution (Fisher, SS290-1) | 99.00 € |
| Nanopipette loading | 20 µl pipette (fisher scientific, 10082012) | 215.00 € | 20 µl pipette (fisher scientific, 10082012) | 215.00 € |
|  | Microloader (fisher scientific, 10289651) | 87.50 € | Microloader (fisher scientific, 10289651) | 87.50 € |
| **Sum 2** (fully operating MoNa setup) |  | **67,407,15** |  | **25,905.62** |


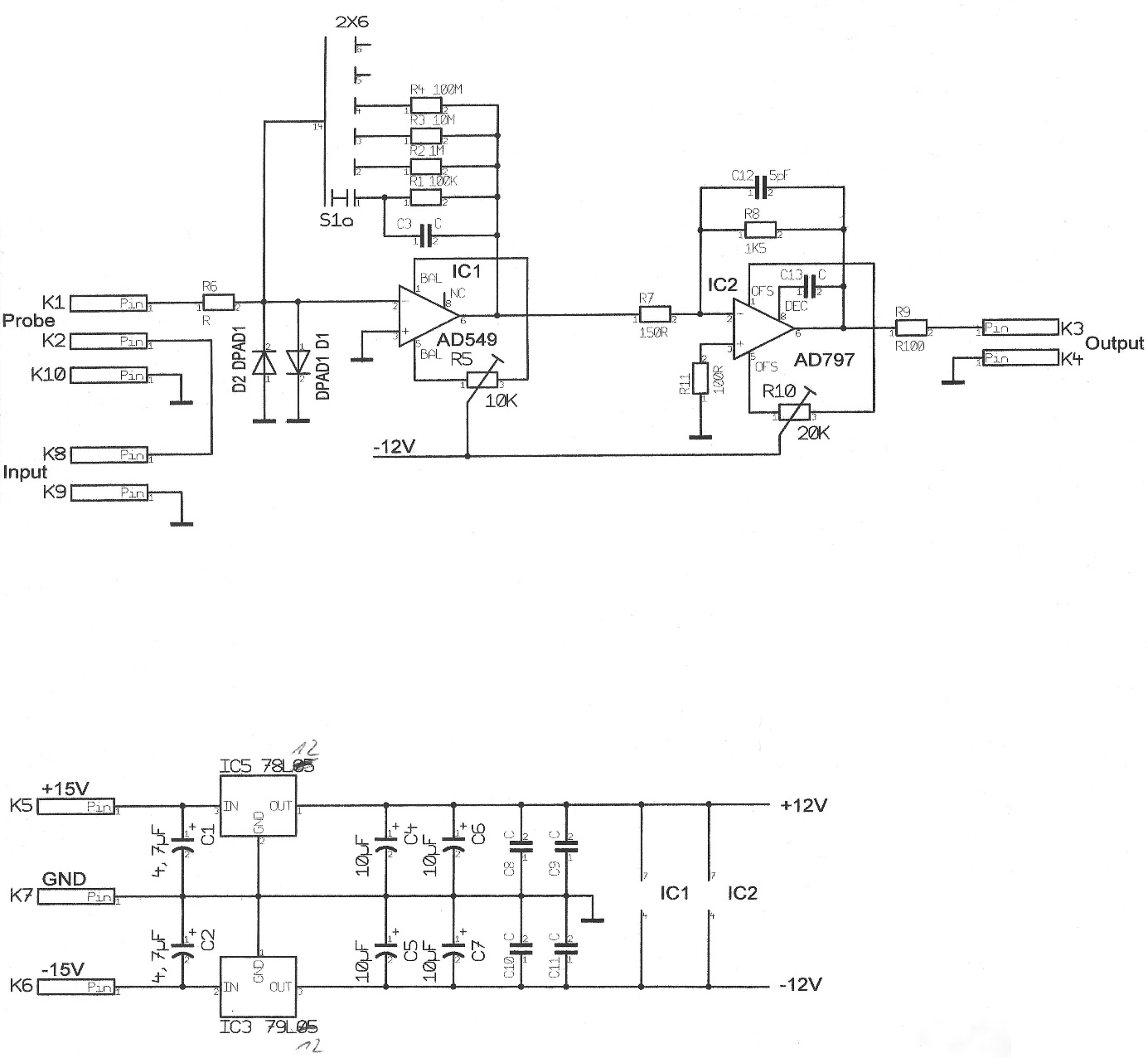


**Figure S9.** Circuit diagram of the operational amplifier. K1 and K2 are connected to the electrodes via alligator clips. K8 and K9 are used to supply voltage to the electrodes. They are connected via BNC (labeled “INPUT”) to the USB A/D converter (Analog-Out 0). K3 and K4 connect to the input of the USB device (Analog-In 0). This output is used to monitor the ion current. The power supply is connected through K5, K6 and K7.


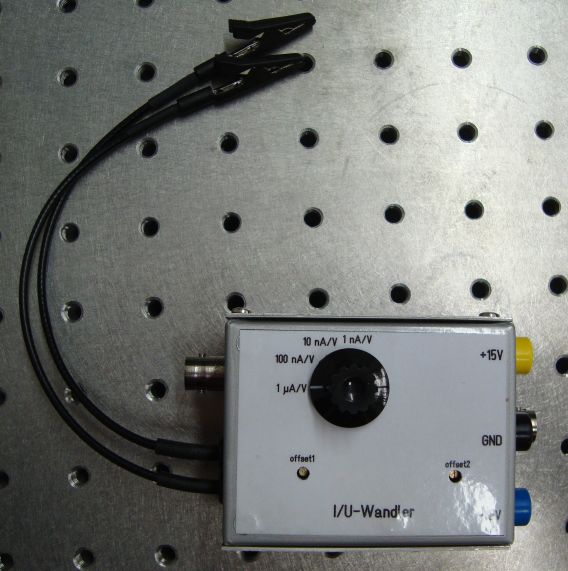


**Figure S10.** Operational Amplifier of the MoNa system. Four selectable current ranges: ±10 nA, 100 nA, 1 µA and 10 µA can be chosen with consequent measurement precision determined by their respective noise levels. 15 V DC are needed to operate the amplifier and can be provided by a power supply or a battery pack for additional portability. The output to the data acquisition interface is scaled over the entire range from -10 V to +10 V, the input range is also scaled from -10 to 10 V. The two black cables are connected to the electrodes.

TECHNICAL DRAWINGS

Technical drawings for the custom built parts of the MoNa system. All drawings are referred to DIN A4. The units of the drawings are given in millimeters [mm]. The diameters of the screws refer to the ISO metric screw thread and are given in [M].

SUPLEMENTARY MOVIES

**Movie S1:** Whitelight tracking of the approach process of the nanopipette towards a cellular membrane. The focal plane was set to the lower region of the cell near the surface. At the beginning of the approach process, the nanopipette is located approx. 20 µm above the focal plane (manual approach) and is only visible as a dark shadow in the background (indicated by the yellow arrow). While the nanopipette approaches the surface of the cell, the tip of the pipette becomes more visible (illuminated ring), till the approach is stopped as the tip reaches the focal plane.

**Movie S2:** Wide-field fluorescence tracking of the labeling process of mitochondria with Mito Tracker Deep Red by nanoinjection. Due continuous nanoinjection it was possible to observe the dynamics of the mitochondria over at least 30 min.

REFERENCES

[1] Simon Hennig, Sebastian van de Linde, Martina Lummer, Matthias Simonis, Thomas Huser, Markus Sauer; “*Instant Live-Cell Super-Resolution Imaging by Nanoinjection of Fluorescent Probes*", Nano Lett., 2015, 15 (2), 1374-1381
